# EssTFNet: integration of adaptive time–frequency and DNA language models for interpretable human essential gene prediction

**DOI:** 10.1093/bib/bbag349

**Published:** 2026-07-03

**Authors:** Dong-Xin Ye, Shi-Shi Yuan, Wei Su, Hong-Qi Zhang, Rui Li, Ye-Chen Qi, Hao Lin, Nanqing Dong, Yan-Ting Jin

**Affiliations:** School of Life Science and Technology, University of Electronic Science and Technology of China, 2006 Xiyuan Avenue, West Hi-Tech Zone, Chengdu 611731, Sichuan, China; Shanghai Innovation Institute, No. 3, Lane 699, Huafa Road, Xuhui District, Shanghai 200231, China; School of Life Science and Technology, University of Electronic Science and Technology of China, 2006 Xiyuan Avenue, West Hi-Tech Zone, Chengdu 611731, Sichuan, China; School of Life Science and Technology, University of Electronic Science and Technology of China, 2006 Xiyuan Avenue, West Hi-Tech Zone, Chengdu 611731, Sichuan, China; School of Life Science and Technology, University of Electronic Science and Technology of China, 2006 Xiyuan Avenue, West Hi-Tech Zone, Chengdu 611731, Sichuan, China; School of Life Science and Technology, University of Electronic Science and Technology of China, 2006 Xiyuan Avenue, West Hi-Tech Zone, Chengdu 611731, Sichuan, China; School of Life Science and Technology, University of Electronic Science and Technology of China, 2006 Xiyuan Avenue, West Hi-Tech Zone, Chengdu 611731, Sichuan, China; School of Life Science and Technology, University of Electronic Science and Technology of China, 2006 Xiyuan Avenue, West Hi-Tech Zone, Chengdu 611731, Sichuan, China; Shanghai Innovation Institute, No. 3, Lane 699, Huafa Road, Xuhui District, Shanghai 200231, China; Shanghai Artificial Intelligence Laboratory, No. 129 Longwen Road, Xuhui District, Shanghai 200232, China; School of Computer Science and Technology, Aba Teachers University, Foshan Road, Shuimo Town, Wenchuan County, Aba Tibetan and Qiang Autonomous Prefecture, Sichuan 623002, China

**Keywords:** human essential gene prediction, DNA language models, deep learning, DeepLIFT, model interpretability

## Abstract

Essential genes are defined as indispensable for an organism’s survival. The loss of function of these genes results in cell death or an inability to complete the normal life cycle. Research on essential genes is pivotal in elucidating the origin and evolution of life, as well as in identifying potential therapeutic targets. Therefore, predicting essential genes is of great scientific importance and has many applications in basic research and the biomedical field. In this study, we propose EssTFNet, a novel, interpretable deep learning framework that combines adaptive time–frequency analysis with a DNA language model to achieve accurate prediction of human essential genes while enabling mechanistic biological interpretation. EssTFNet leverages the architecture of ATFNet, which maps DNA and protein sequences into equivalent time-series signals to extract periodic and nonstationary features, enhancing the model’s capacity to capture complex sequence patterns. Through feature selection and architectural optimization, EssTFNet achieves a favorable balance among prediction accuracy, model interpretability, and cross-tissue generalization. On the S1 benchmark task, EssTFNet outperformed mainstream sequence-based deep learning methods, achieving an area under the curve of 0.9679 and an area under the precision–recall curve of 0.8491. Additionally, the DeepLIFT attribution method was employed to identify functional motifs associated with gene essentiality, offering valuable insights for experimental validation. For the convenience of researchers, we have developed an easy-to-use web server and made it along with the source code in a GitHub repository: https://github.com/QIANJINYDX/EssTFNet. Overall, this study presents a potentially useful methodological framework for human essential genes prediction, which could provide valuable insights for future research and applications in this field.

## Introduction

Essential genes are indispensable to the survival or reproduction of a living organism, playing a crucial role in maintaining cellular life. Studying these genes can help to elucidate the origin and evolution of life, define the minimal gene set required for cellular viability, and infer the genomic architecture and lifestyle of early life forms [1]. Moreover, identifying essential genes in pathogens is key to drug design, as antibiotics primarily target the fundamental metabolic processes of pathogens, making essential genes potential drug targets [2–4]. Similarly, identifying essential genes in cancer cells can aid in discovering novel cancer drug targets [5–7]. Therefore, the identification of essential genes is of substantial scientific significance and broad practical value in both basic research and biomedical fields.

Currently, there are two main approaches for identifying essential genes: experimental and computational methods. Experimental methods can provide specific results for essential genes under different experimental conditions [8], such as RNA interference technology, CRISPR-based genome editing, gene knockout, transposon mutagenesis, and others [9–12]. Although highly reliable, these methods are labor-intensive, costly, and technically demanding, especially in complex organisms [13].

To overcome these limitations, computational methods have been developed to predict gene essentiality using machine learning algorithms [14, 15] and biological feature data [16, 17]. This approach significantly reduces the experimental burden while accelerating the discovery process. For example, in 2005, Chen et al. studied the dispensability of proteins in *Saccharomyces cerevisiae* by combining high-throughput data and machine learning methods [18]. In 2006, Seringhaus et al. reported a machine learning-based method that integrates various intrinsic and predicted features to identify essential genes in the *S. cerevisiae* genome [19]. In 2012, Yuan et al. integrated information-rich genomic features and used three machine learning-based methods to predict lethal knockouts in mice [20]. In 2015, Lloyd et al. analyzed the characteristics of essential genes in the *Arabidopsis thaliana* genome and used *Arabidopsis* as a machine learning-based model to transfer essential annotations to rice and *S. cerevisiae* [21]. In 2017, Guo et al. proposed a method that accurately predicts human essential genes using only nucleotide composition and associated information [22]. In 2019, Campos et al. performed large-scale annotation and exploration of eukaryotic genes and protein functions, predicting essential genes both within and across species using machine learning methods [23]. In 2020, Zhang et al. proposed the DeepHE method, which accurately predicts human essential genes by integrating sequence data and protein–protein interaction (PPI) network features, significantly outperforming traditional machine learning models [24]. In 2023, Li et al. proposed the DeepCellEss framework, which combines convolutional neural networks and self-attention mechanisms to provide interpretable deep learning methods specific to cell lines [25].

While these studies highlight the feasibility of predicting essential genes computationally, several key challenges remain for human essential gene prediction: (i) compared to microbial genomes, the human genome contains much longer and structurally complex sequences, posing significant difficulties for computational modeling [26]. Therefore, it is essential to develop an efficient and accurate long-sequence modeling approach. (ii) Although several DNA language models have been developed [27], there is no model specifically designed for essential genes. Therefore, building a language model focused on essential genes is of great significance. (iii) Current research on human essential gene prediction still lacks in-depth mechanistic analysis, leading to limitations in prediction accuracy and interpretability. How to mine and extract biologically meaningful key features in computational analysis, and improve the algorithm’s ability to understand and utilize data features, is an urgent issue that needs to be addressed. (iv) Most existing gene essentiality prediction models use a binary classification approach, distinguishing genes as either “essential” or “non-essential.” However, in certain task scenarios (e.g. drug target gene screening), gene essentiality often exhibits a continuous variation rather than a simple binary state [28, 29]. Models capable of predicting the degree of essentiality would provide more nuanced insights, enhancing their applicability to drug discovery and personalized medicine [30].

To address these challenges, we propose EssTFNet, an interpretable hybrid framework that fuses feature representations from the adaptive time–frequency model and DNA language model to improve the prediction of human essential genes and their fitness scores. By combining sequence-context embeddings with time–frequency features, EssTFNet improves predictive performance and provides additional clues for interpreting which sequence patterns contribute to the predictions. In our experiments, EssTFNet consistently outperforms existing methods for human essential-gene prediction. Overall, this work provides a practical computational tool for essential-gene prioritization and a mechanistically grounded basis for downstream applications in precision medicine, drug-target discovery, and synthetic biology.

## Materials and methods

**Figure 1:**
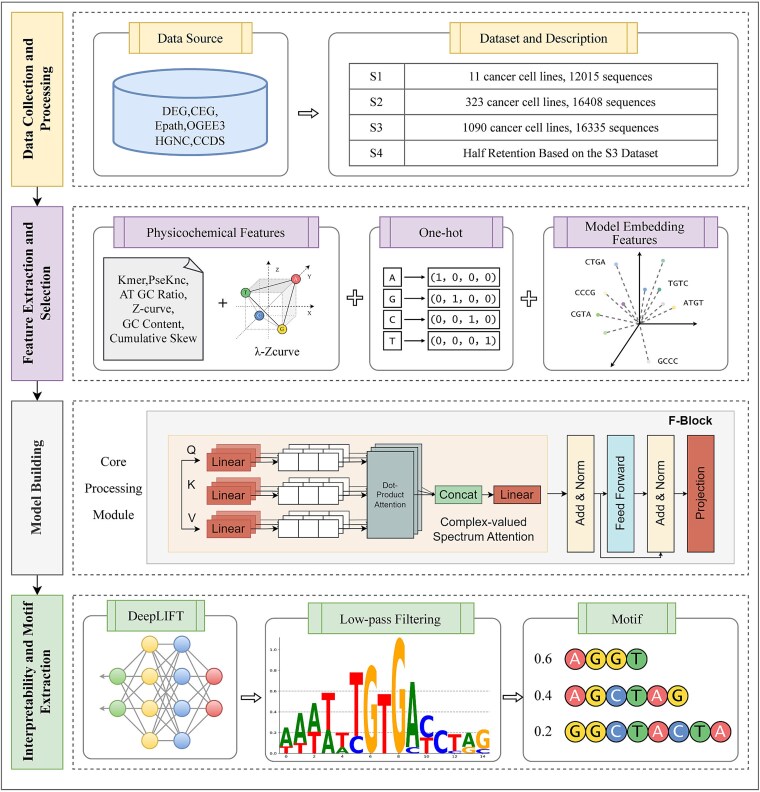
The figure illustrates the workflow of EssTFNet, including data collection and preprocessing, feature extraction and selection, model building, and interpretability analysis.

The overall workflow of EssTFNet is summarized in Fig. 1, including dataset construction, sequence preprocessing, feature extraction, model training, evaluation, and interpretability analysis.

### Data collection and preprocessing

This study utilized a total of five datasets for the analysis. Detailed descriptions of datasets of interest are provided in Table 1.

**Table 1:** Summary of datasets used in this study.

Dataset	Data content	Number of DNA sequences	Coverage	Labels
S1	DNA and protein	12 015	11 cancer cell lines	Binary labels
S2	DNA and protein	16 408	323 cancer cell lines	Binary labels
S3	DNA and protein	16 335	1090 cancer cell lines	Binary labels Fitness scores
S4	DNA and protein	16 335	Same cell lines as S3	Binary labels Fitness scores
DepMap-IT	DNA and protein	875	Pan-Cancer	Binary labels

The S1 dataset used in this study was originally constructed by Guo et al [22] in 2017, which integrates essential gene data from 3 independent studies covering 11 human cancer cell lines (A375, DLD1, GBM, HAP1, HCT116, HeLa, Jiyoye, KBM7, K562, Raji, and RPMI-8226) [6, 31, 32]. The study employed the majority rule method to define core essential genes, whereby a gene was labeled as essential if it was identified as such in more than six cell lines. Gene annotation information was retrieved from the HGNC database, and genes not included in the essential set were labeled as non-essential, forming a well-defined negative sample set [33].

The S2 and S3 datasets are derived from the Project Score initiative and the dataset released by Pacini et al. [34, 35], respectively. The S2 dataset contains 16 408 gene sequences from 323 cell lines, while the S3 dataset contains 16 335 gene sequences from 1090 cell lines.

The S4 dataset was constructed based on the S3 dataset by applying the same majority rule method as in the S1 dataset to define core essential genes. In addition, the regression targets in S4 were defined as the average fitness scores across cancer cell lines in S3, providing a quantitative measure of gene essentiality. This resulted in a more representative subset of essential genes, which was used to build a generalizable essential gene prediction model.

External generalization was assessed using an independent pan-cancer test set constructed from the current public DepMap release, referred to as DepMap-IT [36]. In this study, we used the DepMap Public 26Q1 release downloaded from the DepMap portal, including CRISPR-based gene dependency profiles and cell-line annotation files. Cell line-level CRISPR-based dependency profiles were aggregated across available cancer cell lines to obtain gene-level dependency estimates. Genes with a mean dependency score of at least 0.5 were labeled as essential, whereas genes with a mean dependency score of 0.1 or lower were labeled as non-essential. Genes with intermediate scores were excluded to reduce label ambiguity, and only genes with sufficient valid measurements across cell lines were retained.

To keep the evaluation independent, genes present in the S1, S2, or S3 datasets were removed from the DepMap-derived candidate set. The remaining genes were matched to the DNA and protein sequence annotations required by EssTFNet, and genes without complete sequence information were excluded. This procedure yielded DepMap-IT, a set of 875 genes comprising 51 essential and 824 non-essential genes. DepMap-IT provides a non-overlapping and highly class-imbalanced benchmark for evaluating EssTFNet in a realistic pan-cancer dependency setting.

### Model architecture

**Figure 2:**
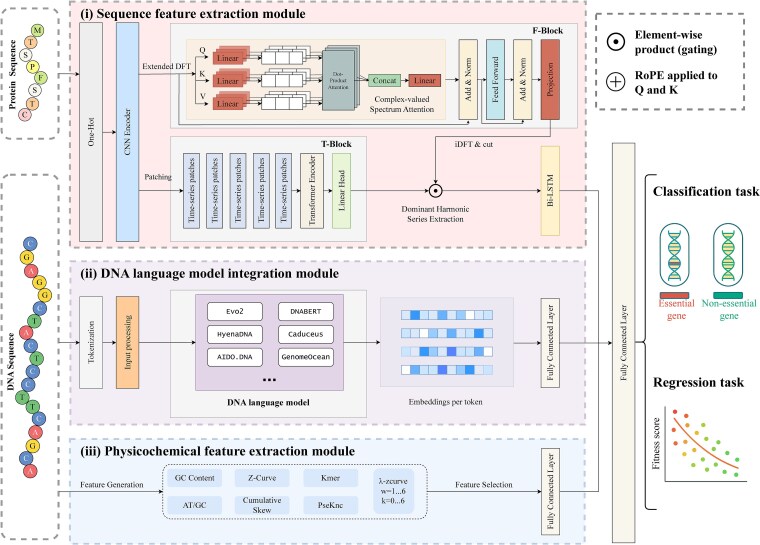
Framework of EssTFNet. EssTFNet has a hybrid architectural design, which efficiently integrates complementary representations from multiple branches. (i) Protein and DNA sequences are first encoded and processed by a CNN (TextCNN) to capture local motif patterns. The resulting features are further modeled by a T-Block for sequential dependencies and an F-Block that performs complex-valued spectrum attention in the time–frequency domain, followed by dominant harmonic series extraction and a Bi-LSTM module. (ii) In parallel, the DNA sequence is fed into a DNA language model to obtain contextual token embeddings, followed by a fully connected layer. (iii) The physicochemical and sequence-derived features of DNA sequence (e.g. GC/AT content, k-mer, Z-curve, skew, PseKNC, etc.) are incorporated into a feature vector through a feature-selection module, followed by a fully connected layer. All feature representations are concatenated and fused through fully connected layers to produce multi-task outputs, including binary essential-gene classification (via a sigmoid activation) and continuous gene essentiality/fitness score regression.

In this study, we designed EssTFNet as a modular architecture to efficiently extract and integrate complementary information from different feature sources. The model consists of three main components: a physicochemical feature branch, a sequence feature branch based on adaptive time–frequency modeling, and a DNA language model branch. The data flow begins with the preprocessing of DNA and protein sequences. DNA and protein sequences are standardized and converted into one-hot encoded representations for sequence-based modeling, while DNA sequences are also used to generate physicochemical feature vectors. These inputs are then fed into the corresponding branches in parallel to extract branch-specific representations. The resulting feature vectors are concatenated and passed through fully connected layers for feature fusion. For binary essential gene prediction, EssTFNet uses a sigmoid-activated classification output to estimate the probability that a gene is essential. For fitness score prediction, an additional regression output is used to predict continuous gene essentiality or dependency scores. The full architecture of the proposed EssTFNet model is schematically illustrated in Fig. 2.

#### Time–frequency network design

When processing DNA and protein sequences, we treat the one-hot encoded sequences as time series and extract both their temporal and frequency information. For extracting this information, we adopted the methods from ATFNet [37]. For an input sequence $S\in{\mathbb{R}}^L$, the T-Block directly processes the sequence in the time domain, generating the output ${S}_o^{(t)}\in{\mathbb{R}}^T$. We use the extended discrete Fourier transform (DFT) to transform $S$ into the frequency domain, generating the extended spectrum $F\in{\mathbb{C}}^{L+T}$. Then, through the inverse discrete Fourier transform (iDFT), the spectrum is converted back to the time domain, and the F-Block generates the output ${S}_o^{(f)}\in{\mathbb{R}}^T$. Finally, the outputs from the T-Block ${S}_o^{(t)}$ and the F-Block ${S}_o^{(f)}$ are combined through a weighted sum to generate the final output $\hat{S}\in{\mathbb{R}}^T$. These weights are derived based on the energy of the fundamental harmonic sequence. The entire process can be formalized as follows:


(1) \begin{equation*} F=\mathrm{Extended}\ \mathrm{DFT}(S) \end{equation*}

(2) \begin{equation*} {\displaystyle \begin{array}{c}{S}_o^{(f)}=\mathrm{F}-\mathrm{Block}(F)\end{array}} \end{equation*}

(3) \begin{equation*} {\displaystyle \begin{array}{c}{S}_o^{(t)}=\mathrm{T}-\mathrm{Block}(S)\end{array}} \end{equation*}

(4) \begin{equation*} {\displaystyle \begin{array}{c}{w}_t,{w}_f=\mathrm{Weighting}(S)\end{array}} \end{equation*}

(5) \begin{equation*} {\displaystyle \begin{array}{c}\hat{S}={w}_t{S}_o^{(t)}+{w}_f{S}_o^{(f)}\end{array}} \end{equation*}

For the extraction of frequency information, we use the extended discrete Fourier transform (Extended DFT). It overcomes the limitations of input length, allowing us to obtain the input spectrum aligned with the DFT frequency bins of the full sequence. The specific formula is as follows:


(6) \begin{equation*} {\displaystyle \begin{array}{c}F\left[k\right]=\sum_{n=0}^{L-1}\kern0.1em x\left[n\right]{e}^{-2\pi i\frac{kn}{L+T}},\ k=0,1,\dots, L+T-1\end{array}} \end{equation*}

By using Equation 6, we obtain a spectrum of length L + T, aligned with the DFT spectrum of the full sequence. For real-valued time series, the conjugate symmetry of the output spectrum is an important feature of the DFT. Utilizing this property, computational cost can be reduced by considering only the first half of the output spectrum, as the second half provides redundant information.

The architecture of the F-Block is based on the Transformer encoder, with all parameters being complex-valued. Specifically, all computations within the F-Block are performed in the complex domain. The F-Block receives the univariate frequency spectrum $F\in{\mathbb{C}}^{\hat{L}}$ generated by the extended DFT as input, where $\hat{L}=\left\lfloor \frac{L+T}{2}\right\rfloor +1$ represents the length of the spectrum.

First, $F$ is normalized by subtracting the mean and dividing by the standard deviation, with the standard deviation calculated from $\mid F\mid \in{\mathbb{R}}^{\hat{L}}$. Subsequently, the mean is added back at the end of the structure. Next, $F\in{\mathbb{C}}^{\hat{L}}$ is embedded into ${F}_d\in{\mathbb{C}}^D$ using a trainable linear projection ${W}_{\mathrm{emb}}\in{\mathbb{C}}^{D\times \hat{L}}$. Since there is less temporal dependence within the frequency domain spectrum, the use of position embeddings is disabled.

Complex-valued spectrum attention. An improved multi-head attention mechanism is used instead of the original attention mechanism. For each head $h=1,2,\dots, H$, the embedded spectrum ${F}_d\in{\mathbb{C}}^D$ is projected onto the frequency dimension $d$ through a trainable projection. Specifically, ${Q}_h={F}_d^T{W}_h^Q,{K}_h={F}_d^T{W}_h^K$,${V}_h={F}_d^T{W}_h^V$, where ${W}_h^Q,{W}_h^K,{W}_h^V\in{\mathbb{C}}^{D\times d}$ are learnable parameters. For each head, complex-valued dot-product attention is performed:


(7) \begin{equation*} {\displaystyle \begin{array}{c}\mathrm{Attention}\left({Q}_h,{K}_h,{V}_h\right)=\mathrm{Softmax}\left(\left|{Q}_h{K}_h^T\right|\right){V}_h\end{array}} \end{equation*}

Then, the final output of the complex-valued spectrum attention is computed as follows:


(8) \begin{equation*} {\displaystyle \begin{array}{c}{\mathrm{head}}_h=\mathrm{Attention}\left({Q}_h,{K}_h,{V}_h\right)\end{array}} \end{equation*}

(9) \begin{equation*} {\displaystyle \begin{array}{c}\mathrm{CSA}\left({F}_d\right)= Concat{\left({head}_1,{head}_2,\dots, {head}_H\right)}^T{W}_0\end{array}} \end{equation*}

where ${W}_O\in{\mathbb{C}}^{hd\times D}$ is a learnable parameter.

Following the design of LayerNorm and FeedForward layers in the Transformer, and extending to the complex domain in the residual connections, the output ${F}_z$ after $M$ layers of encoders is linearly projected to ${F}_o\in{\mathbb{C}}^{\hat{L}}$. Then, we convert ${F}_o$ back to the time domain using the iDFT and take the last $T$ points (the prediction part) as the final output ${X}_o^{(f)}\in{\mathbb{R}}^T$ of the F-Block. The F-Block applies a full complex-valued neural network (CVNN) architecture [38].

The T-Block is responsible for capturing local dependencies in time series, which is more easily handled in the time domain. Chunking is an intuitive and effective method for capturing local dependencies in time series. This chunking is then combined with the Transformer encoder architecture to form the T-Block. First, it splits the input sequence $X\in{\mathbb{R}}^L$ into multiple small chunks ${X}_p\in{\mathbb{R}}^{P\times N}$, where $P$ is the length of each chunk and $N$ is the number of chunks. The small chunks ${X}_p$ are then embedded and fed into the Transformer encoder. Through a linear projection, the final output ${X}_o^{(t)}\in{\mathbb{R}}^T$ is generated.

#### Design of essential gene language model

To incorporate contextual sequence representations learned from large-scale genomic pretraining, we introduced a DNA language model branch into EssTFNet. Before constructing the final framework, we first performed a systematic backbone screening on the S1 dataset to identify a suitable DNA foundation model for human essential gene prediction. The evaluated models included representative k-mer-based, BPE-based, Transformer-based, state-space-based, and long-context genomic language models, including DNABERT, DNABERT-2, DNABERT-S, GROVER, Nucleotide Transformer-v1, Nucleotide Transformer-v2, Nucleotide Transformer-v3, Gena-LM-BERT, HyenaDNA, Caduceus, Evo 2, AIDO.DNA-300M, OmniReg, Genos, and GenomeOcean [39–52].

For each candidate backbone, DNA sequences were processed according to the corresponding tokenizer and input requirements of the original model. The extracted sequence embeddings were then projected through a fully connected layer and integrated with the time–frequency and physicochemical feature branches of EssTFNet. To ensure a fair comparison among different DNA language models, the downstream classifier, training strategy, data partition, and evaluation metrics were kept consistent across all backbone settings.

Based on this screening procedure, the best-performing DNA language model backbone was selected and used as the final language model component in EssTFNet. All DNA foundation models were used with their publicly available pretrained weights.

### Extraction of physicochemical features from DNA sequences

In the process of physicochemical feature extraction, we selected a variety of features, including K-mer, PseKNC features, cumulative skew, Z-Curve, GC content, AT/GC ratio, and λ-Z-curve features, with the λ-Z-curve feature being validated as an effective feature in essential gene studies [22]; see Table 2 for a detailed breakdown of feature types and quantities.

**Table 2:** Feature types and quantities.

Feature type	Number of features
K-mer (*k* = 1,2,3)	84
PseKNC (*k* = 3)	65
Cumulative skew	2
Z-Curve	3
GC content	1
AT/GC ratio	1
λ-Z-curve (w = 1…6, *k* = 0…6)	85 995
Total	86 151

To further optimize the feature set, we applied the recursive feature elimination (RFE) method for feature selection on the S1 dataset. Considering the time overhead that large-scale feature selection might incur, we performed a step-by-step selection for the λ-Z-curve features. Specifically, we divided the 85 995 λ-Z-curve features into 9 groups, each containing 9555 features, and combined them with other features like K-mer, so that the total number of features per group reached 9711. Based on this, we selected the top 1000 features from each group. After merging the 9 groups and removing duplicates, we obtained 8263 features. In the second stage, RFE was applied again to this reduced candidate set to determine the final physicochemical feature subset used for downstream model training. The optimal feature number and the composition of the selected features are reported in the Results section.

### Model interpretability

To enhance the interpretability of our model and understand its internal feature representations and decision-making mechanisms, we employed two methods: t-distributed stochastic neighbor embedding (t-SNE) for feature space visualization and deep learning important features (DeepLIFT) for input attribution analysis. These approaches allow us to investigate how the model transforms and utilizes multilevel features to distinguish essential genes from non-essential ones.

#### Feature representation visualization

To visualize feature representations learned by EssTFNet, we applied t-SNE to branch-level and fused feature embeddings. Specifically, embeddings from the physicochemical feature branch, protein sequence branch, and final fusion layer were projected into 2D space and colored according to essential-gene labels.

t-SNE is particularly effective for visualizing high-dimensional data in a lower-dimensional space (typically two or three dimensions) while preserving local structures. It achieves this by minimizing the Kullback–Leibler (KL) divergence between two probability distributions: one representing pairwise similarities in the high-dimensional space and the other in the low-dimensional embedding. The objective function is given by:


(10) \begin{equation*} {\displaystyle \begin{array}{c}\mathrm{KL}\left(P\parallel Q\right)=\sum_{i\ne j}\kern0.1em {P}_{ij}\log \left(\frac{P_{ij}}{Q_{ij}}\right)\end{array}} \end{equation*}

where ${P}_{ij}$ denotes the similarity between samples $i$ and $j$ in the original feature space, and ${Q}_{ij}$ denotes the corresponding similarity in the embedded space.

By comparing the t-SNE clustering results across different feature representations, we aim to reveal how effectively the model separates different classes or patterns at various stages of processing. This analysis provides valuable insights into the model’s internal feature learning mechanisms and supports further interpretability of its decision-making process.

#### DeepLIFT-based attribution analysis

**Figure 3:**
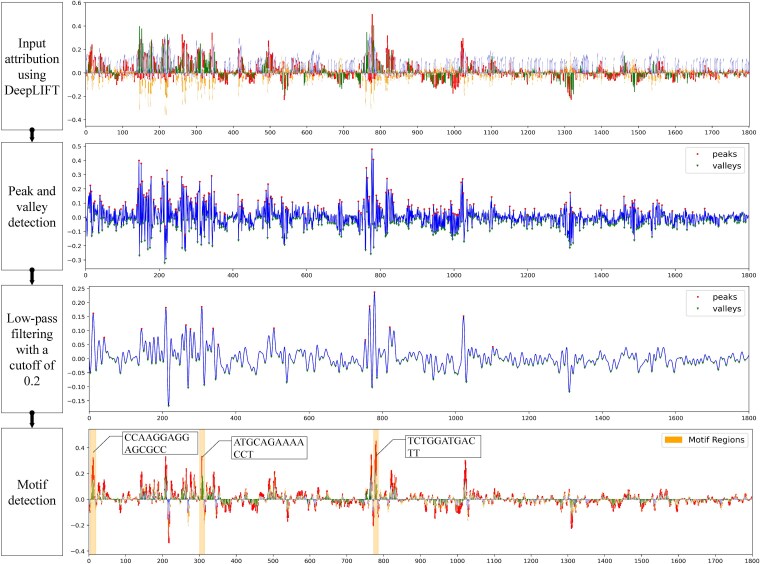
DeepLIFT attribution processing procedure. First, the DeepLIFT algorithm is used for feature attribution on the input sequence, visualizing the DNA sequence feature distribution with a colored fluctuating curve (with the *x*-axis representing the sequence and the *y*-axis representing the attribution strength). Then, key signal nodes are located based on extremum detection (with red/green dots marking peaks and valleys), and the main trend is extracted after smoothing with a low-pass filter at a cutoff frequency of 0.2. Finally, specific binding regions (such as sequences like CCAAGGAGGAGG) are located at the gene sequence level, highlighted by orange annotations.

To explain the model’s prediction decisions for a given DNA sequence based on specific features, we employed the DeepLIFT attribution method [53]. Processing procedure is illustrated in Fig. 3. First, the DNA sequence is standardized to a fixed length of 1800 bp (which corresponds to the average sequence length range), with any insufficient parts filled with “N” characters. Next, based on the background base probability distribution of AGCT in the dataset, we construct the corresponding one-hot form baseline input. The constructed baseline is then inputted into the model along with the actual input, and DeepLIFT is used to calculate the attribution contribution of each position to the prediction result. Without altering the original model structure, this method allows for a refined evaluation of the importance of feature inputs.

It was found that longer motifs are crucial in human essential gene prediction tasks, as these long motifs often correspond to important sequence-specific regulatory elements, whose structural features are significantly correlated with gene functional necessity [54]. Therefore, we integrated digital signal processing techniques into the motif extraction algorithm: by applying a tunable low-pass filter for frequency domain analysis of the sequences, and combining a peak detection algorithm, we achieved precise control over the motif length range.

The backpropagation contribution scores, after signal preprocessing, are fed into a low-pass filter. Unlike a high-pass filter, which emphasizes local mutation features, the low-pass filter systematically suppresses high-frequency noise components through its amplitude-frequency characteristics, while retaining the basic low-frequency features that represent important regulatory patterns. This enables the stable extraction of continuous feature intervals with significant biological meaning from the complex contribution scores. Below is the transfer function of a first-order analog low-pass filter:


(11) \begin{equation*} {\displaystyle \begin{array}{c}H(s)=\frac{1}{\frac{s}{W_n}+1},{W}_n=2\times \frac{f_c}{f_s}\end{array}} \end{equation*}

In this case, ${f}_c$ is the cutoff frequency, and ${f}_s$ is the sampling frequency. The study uses an eighth-order Butterworth low-pass filter to enhance frequency-domain selectivity. The normalized cutoff frequency parameter ${W}_n$ is dynamically configured according to the target motif length: for detecting motifs of lengths 30 bp, 10 bp, and 5 bp, the corresponding threshold values are 0.1, 0.2, and 0.4, respectively.

### Motif screening

To identify sequence motifs significantly associated with essential genes, we performed motif enrichment analysis within a hypergeometric hypothesis testing framework. For each candidate motif, Fisher’s exact test (one-tailed, directionality set for positive enrichment) was applied to a 2 × 2 contingency table (containing motif occurrence counts in positive/negative sample groups and total sequence numbers per group) to assess whether its frequency in the positive sample group significantly exceeded that in the negative group. Raw *P*-values were subsequently adjusted for false discovery rate (FDR) using the Benjamini–Hochberg procedure to mitigate multiple hypothesis testing artifacts [55]. To enhance biological relevance, effect size screening criteria were implemented: odds ratios (ORs) with 95% confidence intervals (CIs) were calculated, retaining only motifs meeting the thresholds of adjusted *P*-value <.05, OR > 2, and CI lower bound >1 as significantly enriched. This dual-filter strategy, integrating statistical significance and effect magnitude thresholds, ensures the identified motifs exhibit both statistical robustness and functional correlation. Detailed results are provided in the model interpretability analysis section of the Results.

### Implementation details

For both classification and regression tasks, we adopted a nested stratified five-fold evaluation strategy. In each outer fold, ~20% of the samples were held out as an independent test set, while the remaining 80% were used as the development set. The development set was further divided into training and validation subsets, corresponding to ~72%, 8%, and 20% of the full dataset for training, validation, and testing, respectively. Stratification was applied in both splitting stages to preserve the original positive-to-negative class distribution. For regression tasks, the same data partitions were used, with stratification based on the binary essential-gene labels and the continuous fitness scores used as regression targets.

To address class imbalance, rebalancing was applied only during classification training. Specifically, minority-class samples were assigned higher sampling probabilities, and a weighted binary cross-entropy loss was used, with the positive-class weight dynamically calculated from the training subset of each fold. Validation and test sets were not resampled and were evaluated under their original class distributions, ensuring that each sample was assessed only once in each fold. Model selection and early stopping were based on validation-set performance, and the final results were reported as the mean and standard deviation across the five independent test folds.

All models were implemented using the Pytorch and Scikit-learn libraries. Training was conducted on a workstation equipped with an Intel(R) Xeon(R) Silver 4310 CPU @ 2.10GHz with 256GB RAM and 4 NVIDIA GeForce RTX 4090D GPUs.

### Evaluation metrics

This study uses multiple evaluation metrics to comprehensively assess model performance. For classification tasks, ACC (accuracy) is used to evaluate overall prediction accuracy, the F1 score is used to address class imbalance by considering both precision and recall, and AUC (area under the ROC curve) and AUPR (area under the PR curve) are used to evaluate the model’s classification ability at different thresholds and its performance on minority classes [56–58]. For regression tasks, the coefficient of determination (*R*^2^) is used to measure the model’s goodness of fit, while the root mean square error (RMSE) quantifies the deviation between predicted and true values. These complementary metrics ensure the comprehensiveness and reliability of the evaluation results from different dimensions.

## Results

### Evaluation of EssTFNet

**Figure 4:**
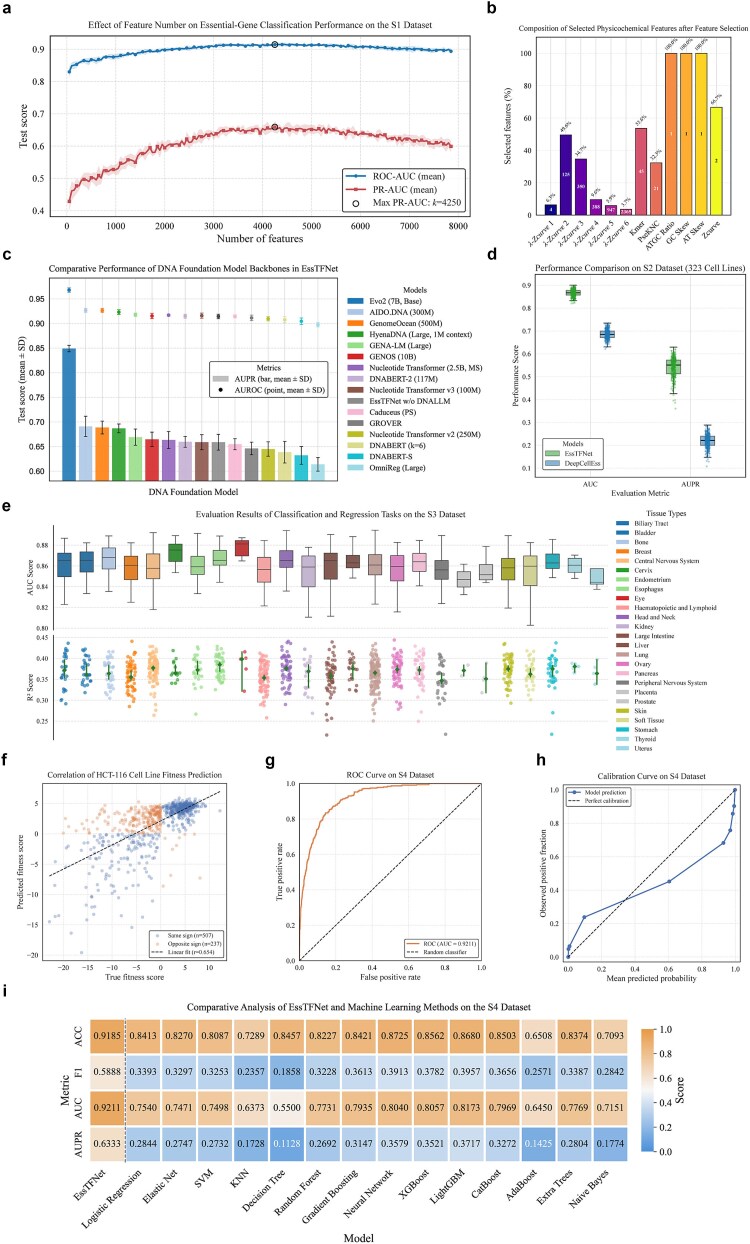
Comprehensive evaluation of EssTFNet across benchmark datasets and prediction settings. (a) Performance of EssTFNet under different numbers of RFE-selected physicochemical features on the S1 dataset. (b) Composition of the final 4250 selected physicochemical features. (c) Benchmarking of different DNA foundation model backbones within the EssTFNet framework on the S1 dataset. (d) Performance comparison between EssTFNet and DeepCellEss on the S2 dataset across 323 cell lines. (e) Classification and regression performance of EssTFNet across tissue contexts in the S3 dataset. (f) Regression prediction analysis on the representative HCT-116 cell line. (g) ROC curve of the S4 general essential-gene prediction model. (h) Calibration curve of the S4 model. (i) Comparison between EssTFNet and conventional machine learning baselines on the S4 dataset.

To determine an appropriate input configuration for EssTFNet, we first performed systematic optimization on the S1 benchmark dataset, focusing on both the dimensionality of the physicochemical feature set and the choice of DNA language model backbone. For physicochemical features, we evaluated model performance under different numbers of RFE-selected features. As shown in Fig. 4a, the PR-AUC increased as informative features were progressively retained, but excessive feature dimensions did not further improve performance and instead introduced potential redundancy. The best performance was achieved when the number of selected features was set to 4250, yielding the highest PR-AUC among all tested feature dimensions. Therefore, we used these 4250 features as the final physicochemical feature representation in subsequent experiments.

We further analyzed the composition of the 4250 selected physicochemical features. As shown in Fig. 4b, λ-Z-curve-derived features constituted the dominant component of the final feature set, accounting for 4179 of the 4250 selected features. In particular, higher-order λ-Z-curve descriptors contributed substantially, including 2365 λ-Z-curve 6 features, 947 λ-Z-curve 5 features, and 388 λ-Z-curve 4 features. This indicates that high-order λ-Z-curve features capture informative nucleotide association patterns that are highly relevant to essential gene prediction. Meanwhile, other feature types, including K-mer, PseKNC, AT/GC ratio, GC skew, AT skew, and Z-curve features, were also retained, suggesting that conventional nucleotide composition and sequence asymmetry descriptors provide complementary information. Together, these results show that the final physicochemical representation is primarily driven by λ-Z-curve features, while still integrating compact descriptors that reflect basic compositional and structural properties of DNA sequences.

We next evaluated whether incorporating DNA foundation model representations could further improve the predictive performance of EssTFNet. To this end, we fixed the optimized 4250-dimensional physicochemical feature set and systematically compared a series of representative DNA foundation model backbones within the same EssTFNet framework, including Evo2, AIDO.DNA, GenomeOcean, HyenaDNA, GENA-LM, GENOS, Nucleotide Transformer, DNABERT-series models, Caduceus, GROVER, and OmniReg. As shown in Fig. 4c, adding DNA foundation model representations consistently improved the model compared with EssTFNet without a DNA language model, indicating that contextual sequence embeddings provide complementary information beyond handcrafted physicochemical descriptors.

Among all evaluated backbones, Evo2 (7B, Base) achieved the best overall performance, with an AUROC of 0.9679 ± 0.0044 and an AUPR of 0.8491 ± 0.0085. In particular, Evo2 produced the highest AUPR among all tested DNA foundation models, suggesting that its sequence representations are especially effective for identifying essential genes under class-imbalanced conditions. Although several other models also achieved relatively high AUROC values, their AUPR scores were lower, indicating that they were less effective in prioritizing positive essential-gene samples. This observation further supports the use of AUPR as a key metric for model selection in this task. Based on these results, Evo2 (7B, Base) was selected as the DNA foundation model backbone in the final EssTFNet configuration.

On the more challenging S2 dataset, EssTFNet consistently outperformed DeepCellEss across 323 cell lines, as shown in Fig. 4d. Specifically, EssTFNet achieved an AUROC of 0.8659 ± 0.0144, substantially higher than DeepCellEss (0.6857 ± 0.0202). A more pronounced improvement was observed in AUPR, where EssTFNet reached 0.5421 ± 0.0434, compared with 0.2185 ± 0.0296 for DeepCellEss. These results indicate that EssTFNet has a stronger ability to identify essential genes across diverse cellular contexts, particularly under class-imbalanced conditions where AUPR provides a more informative assessment of positive-class prediction performance. Overall, the results on S2 further support the robustness and generalizability of EssTFNet for cell line-specific essential gene prediction.

Our model also demonstrated strong adaptability on the larger-scale and tissue-diverse S3 dataset, which included 1090 cell lines and 25 tissue types (Fig. 4e). Across this challenging setting, EssTFNet achieved a mean AUROC of 0.8594 and a mean AUPR of 0.5378, showing performance highly comparable to that on the S2 dataset despite the substantially increased number of cell lines and tissue contexts. Notably, the AUROC decreased by only 0.0065 and the AUPR by only 0.0043 relative to S2, indicating stable discriminative performance under broader cellular heterogeneity. We further evaluated EssTFNet in regression settings to predict continuous fitness values, where the model achieved a mean R^2^ of 0.3660, suggesting that fitness-score prediction remains more challenging than binary classification but that the model can still capture meaningful variation in gene essentiality. On the representative HCT-116 cell line, 507 of 744 genes showed sign-consistent predictions, corresponding to 68.1% of all evaluated genes, while the predicted and true fitness scores exhibited a positive correlation coefficient of 0.654. Genes with higher true fitness scores tended to receive higher predicted scores, as reflected by their distribution along the diagonal trend in Fig. 4f. Together, these results support the robustness of EssTFNet across diverse tissue contexts and highlight its practical potential for ranking and prioritizing functionally important genes in cell line-specific settings.

The ROC curve presents the performance of the general essential gene prediction model (S4) on the test set. The model achieves an AUC of 0.9211, indicating strong discriminatory power between essential and non-essential genes (see Fig. 4g). Notably, this performance surpasses that of the S3 models, which were individually trained for specific cell lines, demonstrating the robustness and generalizability of the unified S4 model across diverse biological contexts. The high true positive rate across a broad range of false positive rates further supports the effectiveness of the model in general essential gene identification. Figure 4h displays the calibration curve of the model on the S4 dataset, assessing the agreement between predicted probabilities and the actual occurrence of positive class instances. Overall, the prediction curve closely follows the ideal calibration line, indicating reliable probabilistic outputs and providing robust support for gene functional studies.

On the S4 dataset, we further compared EssTFNet with a broad set of conventional machine learning methods using four evaluation metrics, including ACC, F1, AUC, and AUPR. As shown in Fig. 4i, EssTFNet achieved the best overall performance across all metrics, with an ACC of 0.9185, F1 score of 0.5888, AUC of 0.9211, and AUPR of 0.6333. In contrast, although several tree-based ensemble methods, such as LightGBM, XGBoost, CatBoost, and Neural Network, obtained relatively competitive ACC and AUC values, their F1 scores and AUPR values remained substantially lower than those of EssTFNet. For example, the best-performing baseline in AUPR reached only 0.3717, and most traditional methods produced F1 scores below 0.40. These results indicate that conventional machine learning models can capture part of the discriminative information from the selected physicochemical features, but they remain limited in identifying essential genes under class-imbalanced conditions. The clear improvements in F1 and AUPR suggest that EssTFNet is more effective in recognizing positive essential-gene samples, rather than simply achieving high overall accuracy. Overall, these findings demonstrate the advantage of integrating DNA foundation model representations with adaptive time–frequency modeling for robust essential gene prediction.

In summary, these experimental findings comprehensively validate EssTFNet’s robust predictive capability derived from its deep feature fusion and spatiotemporal modeling architecture, particularly its exceptional competence in capturing intricate biological feature relationships through combined temporal-frequency domain analysis.

### Model interpretability analysis

#### Adaptive time–frequency analysis of periodic signals

**Figure 5:**
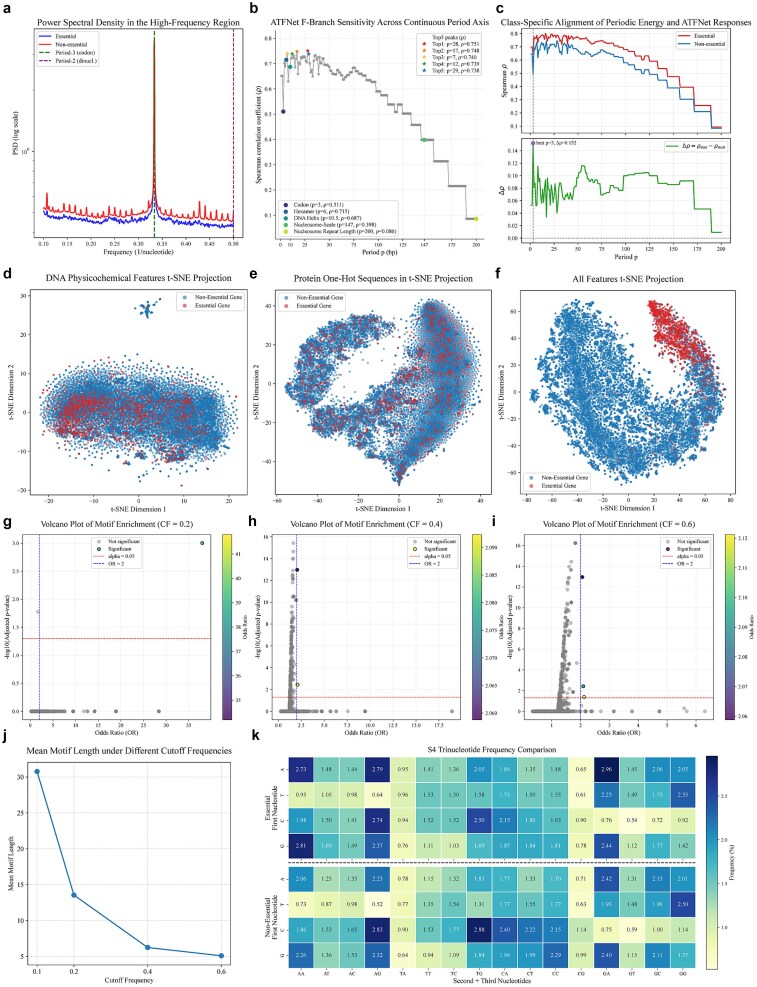
Analyzing and interpreting the model. (a) Power spectral density profiles of essential and non-essential genes. (b) Frequency-response analysis of the ATFNet F-branch across different periodic scales. (c) Class-specific alignment between input periodic energy and ATFNet frequency responses. (d–f) t-SNE embeddings of feature spaces: (d) DNA physicochemical features, (e) protein one-hot sequences, and (F) their combination for essential-gene prediction; each point is a sequence. (g–i) Volcano plots of motif enrichment under dual-validation (Fisher’s exact test + Benjamini–Hochberg). (j) Increasing the Butterworth low-pass cutoff from 0.1 to 0.6 sharply reduces the mean motif length (from >30 bp to ~5 bp), demonstrating a tunable mechanism for extracting motifs of different sizes. (k) Trinucleotide frequency comparison between essential and non-essential genes.

To examine whether the adaptive time–frequency module captures biologically meaningful periodic signals, we analyzed the spectral profiles of essential and non-essential genes and compared them with ATFNet frequency responses (Fig. 5a–c). Power spectral density analysis showed a clear peak near the period-3, or codon-scale, frequency in both essential and non-essential genes (Fig. 5a), consistent with the triplet structure of protein-coding sequences. Non-essential genes showed stronger and more frequent local peaks in the high-frequency region, suggesting greater short-range compositional fluctuation. By contrast, essential genes had a smoother spectral profile. These results suggest that essential and non-essential genes differ not only in sequence composition, but also in the organization of periodic signals.

We then measured the sensitivity of the ATFNet F-branch across a continuous range of periodic scales (Fig. 5b). Model responses were strongly correlated with several biologically relevant or sequence-associated periods, including the hexamer scale ($p=6,\rho =0.715$), the DNA helical scale ($p=10.5,\rho =0.687$), and several longer periodic components. The strongest responses occurred around *P* = 28, 17, 7, 12, and 29 bp. The canonical codon-scale periodicity (*P* = 3) showed a moderate correlation ($\rho =0.511$). This pattern indicates that the F-branch is not limited to the period-3 coding signal, but also captures local and intermediate-range periodic structures that may help distinguish essential genes from non-essential genes.

We further evaluated the class-specific alignment between input periodic energy and ATFNet frequency responses (Fig. 5c). Across most tested periods, essential genes showed higher Spearman correlations than non-essential genes, indicating that the periodic components captured by ATFNet are more closely aligned with the spectral organization of essential genes. The largest difference appeared near the codon-scale periodicity, where the correlation gap reached $\varDelta \rho =0.152$. These results show that ATFNet extracts class-relevant periodic information from DNA sequences and support the use of adaptive time–frequency modeling in EssTFNet. Compared with conventional k-mer statistics, this module describes not only local sequence composition, but also the strength and organization of periodic signals across multiple scales.

#### Feature-space visualization of learned representations

We performed t-SNE dimensionality reduction projection and clustering analysis on various features before model fusion to evaluate the performance of different feature types in distinguishing essential genes from non-essential genes, as shown in Fig. 5d–f. The results show that the DNA physicochemical property features exhibit a clear clustering trend in the 2D space, which can distinguish the two types of gene samples to some extent. This likely reflects intrinsic differences in structural stability, base composition, and other biophysical properties between these two gene categories. In contrast, protein-level features exhibited relatively scattered distributions, with no clear separation observed. After feature integration via model fusion, a comprehensive t-SNE analysis revealed clear category boundaries in the low-dimensional space, confirming the effectiveness of multi-source heterogeneous feature fusion in enhancing discriminative capability. This further indicates that the deep features extracted by the model have a strong capacity to retain class discriminative information, providing a solid foundation for downstream prediction and functional interpretation.

#### Attribution-derived motif enrichment and length control

The identified motifs were filtered using a two-step filtering strategy. Results were visualized through volcano plots, where the *y*-axis represents -log10 (adjusted *P*-value) and the *x*-axis shows OR values on a log2 scale, with reference lines marking FDR thresholds and effect size cutoffs (Fig. 5g–i). Significantly enriched motifs clustered in the upper-right quadrant denote high-confidence positive regulatory elements.

Figure 5j illustrates the relationship between the cutoff frequency and the mean motif length extracted using the Butterworth low-pass filter. As the cutoff frequency increases from 0.1 to 0.6, the mean motif length decreases significantly—from over 30 bp at a cutoff of 0.1 to ~5 bp at a cutoff of 0.6. This trend confirms the effectiveness of the low-pass filtering approach: lower cutoff frequencies allow the extraction of longer motifs by preserving low-frequency signals and filtering out high-frequency noise. Conversely, higher cutoff frequencies retain more high-frequency components, which results in the identification of shorter motifs. These results demonstrate that adjusting the cutoff frequency provides a tunable mechanism for targeting motifs of varying lengths, supporting the dynamic configuration strategy based on expected motif size.

**Figure 6:**
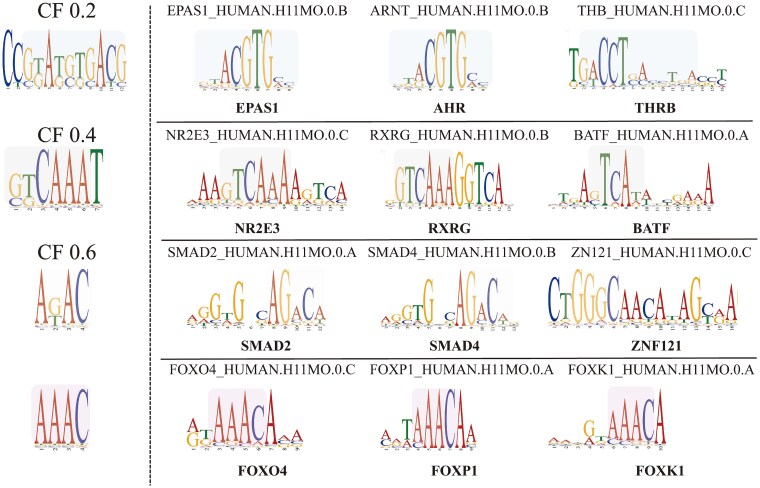
This chart displays multiple human genes and their corresponding motif segments. On the left are essential motifs identified at different CF (cutoff frequency) thresholds, while the right side shows highly similar motif fragments along with their corresponding genes.

To explore differences in nucleotide sequence composition between essential and non-essential genes, we analyzed the frequencies of all 3-mer combinations in the S4 dataset, as shown in Fig. 5k. The results showed that the trinucleotide sequence “AAA” was more frequent in essential genes than in non-essential genes. Because AAA can encode lysine when it occurs within the correct coding frame, this enrichment may partly reflect differences in coding-sequence composition or A-rich sequence patterns between the two gene groups. Lysine-containing regions are often involved in protein–nucleic acid interactions and regulatory processes, including chromatin-related regulation and transcriptional control [59, 60]. However, because the 3-mer analysis did not explicitly model reading frame or codon position, this observation should be interpreted cautiously and should not be taken as direct evidence of lysine residue enrichment. Together with the adenine-rich patterns observed in several motifs in Fig. 6, these results suggest that A-rich sequence features may contribute to the sequence-level signals captured by EssTFNet, providing a preliminary clue for further biological validation.

### Motif analysis

In addition, we performed motif discovery and interpretability analysis to explore sequence patterns associated with essential gene prediction. After parameter optimization, motif screening, and redundancy removal, four nonredundant DNA motifs were retained, including two longer motifs and two shorter motifs, as shown in Fig. 6. To obtain preliminary functional clues, we compared these motifs against known human transcription factor binding profiles in the HOCOMOCO database using Tomtom, and the top three matched transcription factors for each motif are shown on the right side of Fig. 6 [61, 62].

The matched transcription factors included several genes with known roles in important cellular processes. For example, EPAS1 is involved in hypoxia response and angiogenesis, while AHR participates in stress response, immune regulation, and metabolic signaling. In addition, SMAD2 and SMAD4 are key components of the TGF-β signaling pathway, which regulates cell proliferation, differentiation, and apoptosis [63–67]. These associations suggest that the motifs identified by EssTFNet may be related to regulatory programs relevant to cell survival and functional maintenance.

However, these results should be interpreted as hypothesis-generating rather than direct evidence of regulatory causality. Motif similarity analysis indicates potential binding-site resemblance to known transcription factor motifs, but further experimental validation, such as ChIP-seq analysis, reporter assays, or perturbation experiments, would be required to confirm whether these factors directly bind the identified regions and regulate essential genes. Overall, the motif analysis provides interpretable candidate sequence patterns that may help guide future biological validation of essential gene regulatory mechanisms.

### Generalization and context-specific transfer analysis

#### External validation on DepMap-IT

We examined the generalization ability of EssTFNet from three complementary perspectives: external pan-cancer testing, transfer to condition-specific dependency and fitness prediction, and tissue-level discrimination of essentiality patterns. This design allowed us to evaluate whether the model only performs well on the training-related setting or can also retain useful predictive signals across independent datasets, perturbation contexts, and tissue backgrounds.

**Figure 7:**
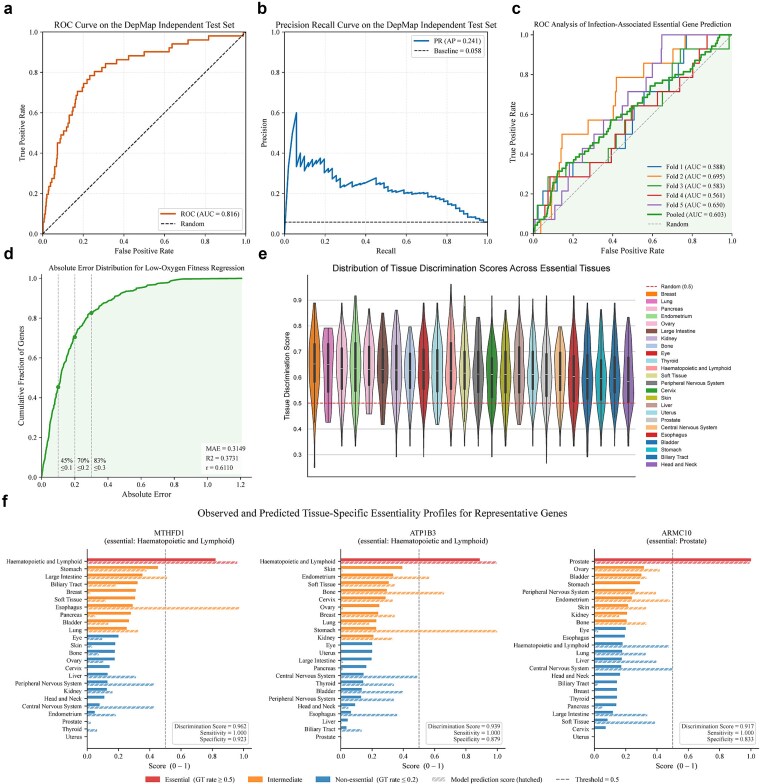
External generalization, condition-specific transfer, and tissue-level essentiality analysis of EssTFNet. (a) ROC curve of EssTFNet on the independent DepMap-IT pan-cancer test set, showing external discriminative performance under a realistic class-imbalanced setting. (b) Precision–recall curve on the same DepMap-IT test set. The dashed line indicates the baseline positive rate. (c) ROC curves for infection-associated dependency prediction across five fine-tuning folds. The S4-trained EssTFNet model was used as initialization and adapted to an infection-associated CRISPR screening dataset. (d) Cumulative absolute-error profile for low-oxygen fitness-score regression. Vertical dashed lines indicate the proportions of genes with absolute prediction errors below 0.1, 0.2, and 0.3. (e) Distribution of tissue discrimination scores across 24 tissue types. Each violin represents the score distribution of tissue-specific genes within one tissue, and the dashed horizontal line denotes the random baseline of 0.5. (f) Representative gene-level tissue-specific prediction examples for MTHFD1, ATP1B3, and ARMC10, respectively. Solid bars indicate observed tissue-level essentiality status, whereas hatched bars indicate EssTFNet prediction scores across tissues.

External generalization was first assessed using DepMap-IT, an independent pan-cancer test set that was not used during model development. On this highly class-imbalanced dataset, EssTFNet achieved an AUROC of 0.816 (Fig. 7a), indicating that the model maintained good discriminative performance in an external DepMap-derived setting. The precision–recall curve further showed an average precision of 0.241, which was substantially higher than the baseline positive rate of 0.058 (Fig. 7b). These results suggest that EssTFNet can prioritize essential genes more effectively than random ranking under realistic class-imbalanced conditions.

#### Condition-specific transfer analysis

We next evaluated whether the representation learned by EssTFNet could be adapted to condition-specific dependency prediction. For this purpose, we used an infection-associated CRISPR screening dataset, which differs from general essential-gene prediction in both biological context and label definition. The S4-trained EssTFNet model was used as initialization and fine-tuned on the infection-associated data. Genes showing significant positive or negative selection under infection conditions were labeled as infection-associated dependency genes, whereas genes without significant signals in either direction were labeled as non-hit genes. Ambiguous or conflicting genes were excluded to reduce label noise. This procedure produced 4210 labeled genes, including 70 positives and 4140 negatives, while 15 303 genes were treated as uncertain and excluded from evaluation. The resulting positive rate was only 1.66%, making this a strongly class-imbalanced and challenging transfer setting.

As shown in Fig. 7c, EssTFNet achieved a pooled AUROC of 0.603 across five folds, with fold-level AUROC values ranging from 0.561 to 0.695. Although this performance was lower than that observed for general essential-gene prediction, the model remained above the random baseline in most folds. This result indicates that part of the essential-gene representation learned by EssTFNet can be transferred to infection-associated dependency prediction. At the same time, the modest performance also highlights the difficulty of modeling condition-specific dependencies when positive samples are scarce.

**Table 3:** Ablation study of EssTFNet modules on prediction performance.

**Model name** ^a^	**ACC**	**F1**	**AUC**	**AUPR**
**EssTFNet**	**0.9380 ± 0.0057**	**0.7637 ± 0.0154**	**0.9679 ± 0.0044**	**0.8491 ± 0.0085**
Feature-only	0.8918 ± 0.0059	0.6050 ± 0.0186	0.9137 ± 0.0060	0.6385 ± 0.0133
No ATFNet	0.9354 ± 0.0079	0.7436 ± 0.0355	0.9585 ± 0.0114	0.8003 ± 0.0309
No DNA LLM	0.8930 ± 0.0182	0.6064 ± 0.0169	0.9134 ± 0.0039	0.6481 ± 0.0136
No protein	0.9365 ± 0.0085	0.7610 ± 0.0173	0.9668 ± 0.0042	0.8466 ± 0.0066
No DNA LLM + No Protein	0.8993 ± 0.0125	0.6011 ± 0.0112	0.9126 ± 0.0053	0.6518 ± 0.0108

Bold values indicate the best performance for each metric. ^a^EssTFNet is the full model with Evo2-7B-base as the DNA language-model backbone. Feature-only uses only selected physicochemical features. No ATFNet removes the adaptive time–frequency module. No DNA LLM removes the Evo2-7B-base branch. No Protein removes the protein sequence branch. No DNA LLM + No Protein removes both the Evo2-7B-base branch and the protein sequence branch.

We further considered whether EssTFNet could be extended from binary prediction to condition-specific regression. The model was evaluated on a low-oxygen fitness prediction task derived from a genome-wide CRISPR growth screen conducted under three oxygen concentrations, 21%, 5%, and 1%. This screen identified genes with oxygen-dependent fitness effects. Because this task requires continuous score prediction rather than binary essential-gene classification, EssTFNet was initialized with the S4-trained weights and fine-tuned to predict low-oxygen fitness scores. As shown in Fig. 7d, the model achieved an MAE of 0.3149, an ${R}^2$ of 0.3731, and a Pearson correlation coefficient of 0.6110. The cumulative absolute-error distribution showed that 45% of genes had an absolute prediction error below 0.1, 70% below 0.2, and 83% below 0.3. These results show that EssTFNet captures a meaningful trend in low-oxygen fitness variation, although accurate point-wise regression remains challenging. The analysis therefore supports the transferability of EssTFNet from general essential-gene prediction to condition-specific fitness regression.

#### Tissue-level and gene-level essentiality discrimination

Beyond external and perturbation-based transfer, we also asked whether EssTFNet can capture tissue-specific essentiality patterns. The S3 dataset was analyzed at the tissue level by aggregating binary essentiality labels across cell lines within each tissue. For each gene, this aggregation estimated the fraction of cell lines in which the gene was essential in a given tissue. Based on the resulting cross-tissue profiles, tissue-specific genes were defined as genes that were essential in at least one tissue but largely non-essential in at least one other tissue. This procedure identified 610 tissue-specific genes for downstream analysis.

For each tissue-specific gene, we computed a tissue discrimination score. This score measures whether the model assigns higher prediction scores to tissues in which a gene shows high essentiality and lower scores to tissues in which the same gene shows low essentiality. A higher score, therefore, reflects stronger discrimination of tissue-dependent essentiality.

After filtering tissue types with insufficient tissue-specific genes, 24 tissue types were retained for tissue-level discrimination analysis. As shown in Fig. 7e, tissue discrimination scores were consistently above the random baseline of 0.5 across all 24 tissues. The overall median score was ~0.615, indicating that EssTFNet captures tissue-dependent essentiality signals beyond random prediction. Median scores ranged from ~0.583 to 0.653 across tissues. Breast, lung, and pancreas showed relatively higher median scores, whereas head and neck, biliary tract, and stomach showed lower values. The number of tissue-specific genes also varied substantially across tissues, ranging from about 28 to 230. Despite this variation, the score distributions remained consistently above the random baseline, suggesting that EssTFNet retains tissue-level discriminative ability across diverse biological contexts.

We then selected three representative genes to examine tissue-specific prediction behavior at the individual-gene level (Fig. 7f). For each gene, the observed tissue-level essentiality profile was compared with the corresponding EssTFNet prediction scores. Solid bars denote the observed essentiality status across tissues, whereas hatched bars denote model-predicted scores. Tissues with observed essentiality rates above 0.5 were treated as essential contexts, and tissues with rates below 0.2 were treated as non-essential contexts.

For MTHFD1, the observed profile showed a clear essentiality signal in hematopoietic and lymphoid tissues. EssTFNet assigned a high prediction score to this tissue while keeping scores generally lower in most non-essential tissues, yielding a discrimination score of 0.962, a sensitivity of 1.000, and a specificity of 0.923. ATP1B3 showed a similar pattern and was also essential in hematopoietic and lymphoid tissues. The model correctly identified this essential tissue context and achieved a discrimination score of 0.939, although several intermediate tissues also received relatively high scores, suggesting partial context similarity or residual uncertainty. For ARMC10, which showed prostate-specific essentiality, EssTFNet assigned the highest prediction score to prostate tissue and achieved a discrimination score of 0.917, with a sensitivity of 1.000 and a specificity of 0.833.

These representative cases show that EssTFNet can recover tissue-specific essentiality patterns at the individual-gene level. Rather than only estimating whether a gene is globally essential, the model assigns higher scores to tissues in which the gene is more likely to be essential and lower scores to non-essential tissue contexts. Together, the external test, condition-specific transfer tasks, and tissue-level analyses indicate that EssTFNet learns transferable sequence-based representations that remain informative across independent datasets, perturbation conditions, and tissue backgrounds.

### Ablation study

To investigate the contribution of each component in EssTFNet, we conducted a comprehensive ablation study, as summarized in Table 3. The complete EssTFNet model achieved the best overall performance across all evaluation metrics, with an ACC of 0.9380 ± 0.0057, F1 score of 0.7637 ± 0.0154, AUC of 0.9679 ± 0.0044, and AUPR of 0.8491 ± 0.0085. These results demonstrate the effectiveness of the fully integrated architecture.

When only the selected physicochemical features were used, the Feature-only model showed a clear performance decrease, with the AUPR dropping to 0.6385 ± 0.0133 and the F1 score decreasing to 0.6050 ± 0.0186. Similarly, removing the DNA language model branch led to a substantial reduction in performance, with the No DNA LLM model achieving an AUPR of 0.6481 ± 0.0136. The comparable performance of Feature-only and No DNA LLM indicates that the Evo2-based DNA language model provides essential contextual sequence representations that substantially enhance the prediction of essential genes.

**Figure 8:**
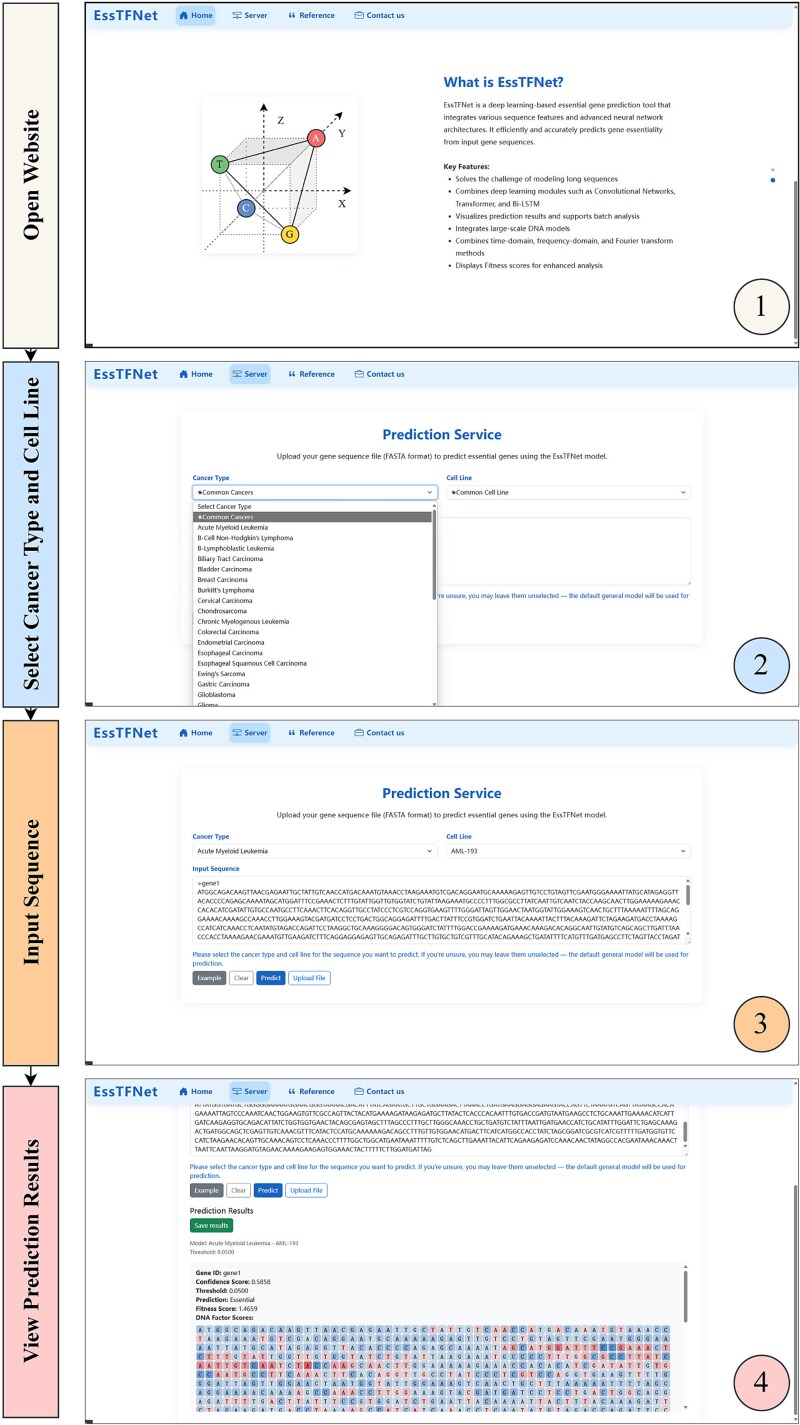
Step-by-step guide for essential gene sequence prediction. (1) Open the EssTFNet web interface. (2) Navigate to the prediction service page and select the cancer type and cell line. (3) Paste or upload the gene sequence (FASTA format) and submit the query. (4) View the predicted result and the corresponding score/visualization outputs on the results panel.

We further assessed the effect of the adaptive time–frequency module. Removing ATFNet resulted in consistent decreases across all metrics, particularly in AUPR, which declined from 0.8491 ± 0.0085 to 0.8003 ± 0.0309. This suggests that the adaptive time–frequency module contributes complementary information by capturing periodic and nonstationary sequence patterns. In contrast, removing the protein branch caused only a slight decrease in performance, with the No Protein model achieving an AUPR of 0.8466 ± 0.0066, indicating that protein sequence information provides additional but relatively modest complementary benefits. When both the DNA language model and protein branches were removed, performance remained substantially lower than that of the full model, further confirming the importance of multimodal feature integration. Overall, these ablation results validate the necessity of combining selected physicochemical features, DNA foundation model representations, adaptive time–frequency modeling, and protein sequence information within EssTFNet.

### Web server

To enhance the accessibility and usability of EssTFNet, we developed a fully featured and user-friendly web server (https://esstfnet.art/). This platform integrates essential gene prediction models for 1090 different human cell lines, based on EssTFNet, and aims to provide researchers with convenient, fast, and high-precision prediction services. For the benefit of the community and researchers, we have made our source code openly available through a public repository: https://github.com/QIANJINYDX/EssTFNet. The models and datasets are also accessible at: https://zenodo.org/records/15552651.

The workflow is illustrated in Fig. 8. Users simply need to upload or enter the target DNA sequence and select the corresponding cell line from the dropdown menu to perform model predictions online. In cases where specific cell line information is unavailable, the platform also offers a general-purpose model suitable for preliminary analysis and exploratory studies across diverse experimental conditions. Upon submission, prediction results are automatically generated and presented in a dedicated results panel. In addition to the predicted gene scores, the server also integrates the DeepLIFT algorithm to assess the impact of each base pair on the model’s prediction results. This attribution analysis helps users understand which regions of the sequence are most critical to the model’s judgment, providing important references for further functional annotation and experimental design. The platform provides strong computational support for gene function research, target screening, and mechanism exploration, serving as an important bridge between artificial intelligence and functional genomics research.

## Discussion

Although the experimental results show that EssTFNet achieves a favorable balance among prediction accuracy, model interpretability, and cross tissue generalization, we acknowledge that the current framework still has several limitations. EssTFNet mainly focuses on DNA sequence modeling and has improved the use of DNA information through time frequency mapping and integration with DNA language model representations. However, its attribution and mechanistic analyses do not yet fully incorporate protein-level sequence and structural information. Important biological features, including conserved amino acid regions, secondary structures, and functional sites in translated proteins, remain to be systematically integrated [68].

Therefore, future work will focus on the following directions: (i) cross-modal modeling: introduce protein sequence and structural information to enable end-to-end modeling from transcription to translation by integrating DNA and protein language models. (ii) Multi-omics data integration: incorporate multi-omics data such as transcriptomics and epigenomics into model training to enhance predictive performance and generalization under specific biological contexts. (iii) Interpretability mechanism optimization: further incorporate graph neural networks (GNNs) and structural attribution methods to improve the model’s interpretability at the spatial structural level. (iv) Expansion of task applicability: explore the applicability of EssTFNet to other gene function prediction tasks, such as noncoding RNA function prediction [69] and cancer driver gene identification, to enhance its generality and practicality.

In summary, EssTFNet provides an efficient, interpretable, and extensible new framework for sequence-based functional gene identification. By integrating multimodal information and structural knowledge in the future, it holds great promise for advancing the application of deep learning methods in gene function modeling.

## Conclusion

In this study, we propose a novel and highly interpretable deep learning framework EssTFNet, which integrates time–frequency domain feature extraction mechanisms with a DNA language model to achieve high-precision prediction and in-depth mechanistic analysis of essential genes. EssTFNet effectively leverages both the structural characteristics and semantic information of DNA sequences, demonstrating superior performance in capturing long-range dependencies and identifying key functional segments. The main contributions of this work are summarized as follows: (i) introduction of the ATFNet architecture: we innovatively map DNA sequences into equivalent time-series signals and extract both periodic and nonstationary features through an adaptive time–frequency analysis module. This enhances the model’s ability to capture long-range dependencies and complex sequence patterns, significantly improving performance in processing long sequences. (ii) To use contextual information from genomic sequences, we integrated an Evo2-7B-base DNA foundation model backbone into EssTFNet. This branch provides sequence-level representations that complement the adaptive time–frequency and physicochemical feature modules. (iii) Introduction of a multi-scale interpretability mechanism to analyze model decision basis: to enhance the biological interpretability of the model, we employ the DeepLIFT attribution method for systematic analysis of the model’s outputs. Combined with low-pass filter design, this approach identifies and extracts important functional motifs at different scales. It not only reveals the key regions the model focuses on, but also provides a theoretical foundation for subsequent functional annotation and experimental validation.

**Key Points**

- We map nucleotide sequences to equivalent time-series signals to capture periodic and nonstationary patterns that conventional sequence models often miss.
- Through feature selection and architectural optimization, EssTFNet balances accuracy, interpretability, and cross-tissue generalization.
- On the S1 benchmark, EssTFNet achieved AUROC = 0.9679 and AUPR = 0.8491, while the S4 general model reached AUROC = 0.9211 and AUPR = 0.6333.
- Using DeepLIFT attribution, we identify functional motifs associated with gene essentiality, offering testable hypotheses for experimental validation.
- For community use, we provide an easy-to-operate web server and open-source code (GitHub: https://github.com/QIANJINYDX/EssTFNet).

## Data Availability

The source code is openly available through a public repository: https://github.com/QIANJINYDX/EssTFNet.
